# Once-weekly insulins: a promising approach to reduce the treatment burden in people with diabetes

**DOI:** 10.1007/s00125-024-06158-9

**Published:** 2024-04-29

**Authors:** Roberto Trevisan, Matteo Conti, Stefano Ciardullo

**Affiliations:** 1 Department of Medicine and Surgery, University of Milano Bicocca, Milan, Italy; 2 Endocrine and Diabetology Unit, Azienda Socio Sanitaria Territoriale Papa Giovanni XXIII, Bergamo, Italy; 3 Department of Medicine and Rehabilitation, Policlinico di Monza, Monza, Italy

**Keywords:** Adherence, BIF, Icodec, Insulin, Review, Weekly

## Abstract

Despite the availability of new classes of glucose-lowering drugs that improve glycaemic levels and minimise long-term complications, at least 20–25% of people with type 2 diabetes require insulin therapy. Moreover, a substantial proportion of these individuals do not achieve adequate metabolic control following insulin initiation. This is due to several factors: therapeutic inertia, fear of hypoglycaemia and/or weight gain, poor communication, complexity of insulin titration, and the number of injections needed, with the associated reduced adherence to insulin therapy. Once-weekly insulins provide a unique opportunity to simplify basal insulin therapy and to allow good glycaemic control with a low risk of hypoglycaemia. Several approaches to developing a stable and effective once-weekly insulin have been proposed, but, to date, insulin icodec and basal insulin Fc (insulin efsitora alfa) are the only two formulations for which clinical studies have been reported. The results of Phase I and II studies emphasise both efficacy (in term of glucose levels) and potential risks and adverse events. Phase III studies involving insulin icodec are reassuring regarding the risk of hypoglycaemia compared with daily basal insulin analogues. Despite some concerns raised in ongoing clinical trials, the available data suggest that weekly insulins may also be an option for individuals with type 1 diabetes, especially when adherence is suboptimal. For the first time there is an opportunity to make an important breakthrough in basal insulin therapy, particularly in people with type 2 diabetes, and to improve not only the quality of life of people with diabetes, but also the practice of diabetologists.

**Graphical Abstract:**

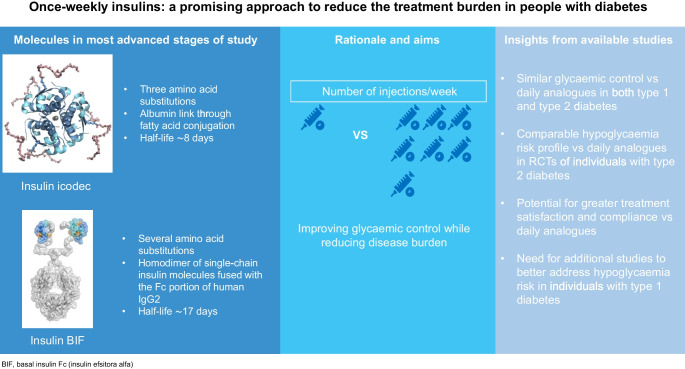

**Supplementary Information:**

The online version contains a slideset of the figures for download available at 10.1007/s00125-024-06158-9.

## Introduction

According to the 2021 IDF diabetes atlas, 536 million adults worldwide are living with diabetes, a number that is expected to increase to 783 million by 2045 [1]. The clinical and economic burdens of the diabetes pandemic are related to chronic micro- and macrovascular complications of the disease and have an enormous impact on both individuals with diabetes and healthcare systems. It has been known for decades that achievement of good glycaemic control can significantly reduce the incidence of complications in both type 1 and type 2 diabetes [2, 3]. Moreover, cardiovascular outcome trials conducted in the last few years in participants with type 2 diabetes have robustly shown how the use of specific drugs that produce weight loss (i.e. sodium–glucose cotransporter 2 inhibitors and glucagon-like peptide 1 receptor agonists) can provide protection against adverse cardiovascular and renal outcomes, independently, at least in part, from glucose control [4, 5]. Based on these remarkable results, recent guidelines suggest the use of these two classes of drugs (together with metformin) as first- or second-line agents in most individuals with type 2 diabetes [6].

Nonetheless, it is well known that deterioration in glycaemic control is common during the natural history of type 2 diabetes [7]. In a recent analysis of data from the USA, the proportion of individuals with type 2 diabetes meeting the HbA_1c_ target of <53 mmol/mol (<7%) not only did not improve, but also declined from 57.4% in 2007–2010 to 50.5% in 2015–2018 [8]. It is therefore conceivable that insulin, besides being the only treatment option for those with type 1 diabetes, will continue to play a prominent role in the management of type 2 diabetes. From its discovery in 1921, enormous progress has been made in the field of insulin therapy [9]. Basal insulins, most of which are administered once daily, are generally preferred over fast-acting insulin analogues in individuals with type 2 diabetes, as they lead to similar glycaemic control with a much lower risk of hypoglycaemia and higher patient satisfaction [10]. In the last few years, insulin degludec and insulin glargine U300 have shown clear superiority over insulin glargine U100 in terms of risk of hypoglycaemia [11, 12]. However, insulin treatment is frequently delayed and started only in cases of severe hyperglycaemia, and, when initiated, only a fraction of people achieve good glycaemic control. For instance, in a real-world study including individuals starting either degludec or glargine U300, baseline HbA_1c_ was 82 mmol/mol (9.7%) and only a quarter of participants achieved an HbA_1c_ <53 mmol/mol (7.0%), with similar results between the two analogues [13]. There may be several reasons for these results, including therapeutic inertia, fear of hypoglycaemia and/or weight gain, poor communication between the patient and the physician, and treatment complexity [14]. An online survey has shown that people with type 2 diabetes generally have a positive attitude towards once-weekly glucose-lowering medications, particularly among injection users [15]. In this context, it is conceivable that once-weekly basal insulins might achieve higher adherence and patient satisfaction than daily insulin, leading to better glycaemic control, provided that they do not lead to increased rates of hypoglycaemia.

In the present review we provide an overview of the weekly insulin analogues that are currently being studied for the treatment of both type 1 and type 2 diabetes, focusing on the two molecules that are furthest along the clinical experimental programme: insulin icodec and basal insulin Fc (BIF; insulin efsitora alfa). It should be noted that the data available for these new weekly insulins are derived from clinical trials designed by the manufacturers and are not necessarily applicable to those people with diabetes who are not represented by the participants enrolled in the trials. Other weekly insulin formulations have also been proposed, but they are still in an early stage of development or have shown excessive variability in absorption and efficacy. Useful information in this regard can be found in recent reviews [9, 16].

## Once-weekly insulins

### Pharmacokinetic considerations

**Fig. 1:**
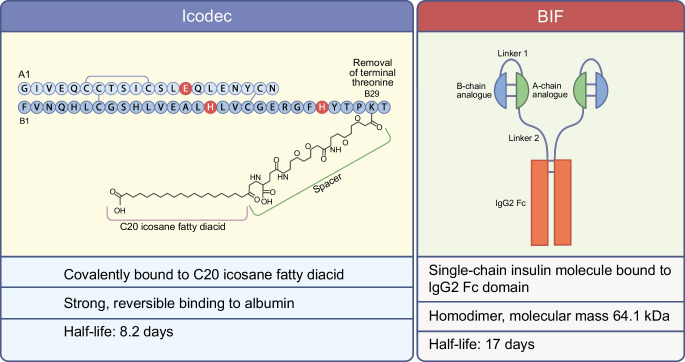
Molecular and pharmacokinetic features of icodec and BIF. The icodec molecule is characterised by three amino acid substitutions compared with human insulin (shown in red) and attachment to a C20 icosane fatty diacid, allowing strong, reversible binding to albumin and resulting in a half-life of 8.2 days. The BIF molecule consists of a homodimer of single-chain insulin molecules covalently fused with the Fc portion of human IgG2. This molecule has a half-life of 17 days. This figure is available as part of a downloadable slideset

As most peptides have a relatively short half-life, substantial structural changes to the insulin molecule are required to achieve the pharmacokinetic properties necessary to ensure a flat profile for at least 1 week. The ways in which this has been achieved for icodec and BIF are very different (Fig. 1).

The icodec molecule is characterised by three amino acid substitutions and, similarly to degludec, attachment to a C20 icosane fatty diacid. The amino acid substitutions are aimed at reducing enzymatic degradation, decreasing the affinity for the insulin receptor (therefore reducing insulin receptor-mediated clearance) and improving solubility. The fatty acid molecule, on the other hand, allows strong, non-covalent reversible binding to albumin, thereby forming an inactive circulating depot from which insulin molecules are continuously and slowly released [17, 18]. These changes translate into a half-life of 8.2 days, with dose-proportional concentrations being achieved [18]. A recent study also showed that total icodec exposure was similar after s.c. injection of icodec in the thigh, abdomen and upper arm [19].

In contrast, BIF is based on IgG–Fc fusion technology, similar to drugs such as etanercept and dulaglutide [20]. In this case, a homodimer of single-chain insulin molecules is covalently fused with the Fc portion of human IgG2. IgGs, along with albumin, are the plasma proteins with the longest half-lives (approximately 20 days). Several amino acid substitutions minimise insulin self-association, confer chemical and physical stability and reduce insulin receptor affinity [21]. Dose–response profiles have been demonstrated in both rats and humans, with a half-life of 17 days and an almost flat profile [22].

### Data from Phase II randomised clinical trials

#### Insulin icodec

In the Phase II clinical trial programme, icodec was compared with glargine U100 in both insulin-naive and insulin-experienced individuals with type 2 diabetes. The first published trial was a 26 week double-blind, double-dummy RCT including 247 insulin-naive participants with type 2 diabetes inadequately controlled [HbA_1c_ 53–80 mmol/mol (7.0–9.5%)] on metformin with or without a dipeptidyl peptidase-4 inhibitor (DPP4i) [23]. The primary endpoint was change in HbA_1c_ at week 26. Starting doses were 70 U/week for icodec and 10 U/day for glargine 100U. Insulin dose adjustments were made once a week, based on the three preceding fasting glucose measurements, with a target of 3.9–6.0 mmol/l in both arms. The mean reductions in HbA_1c_ were –14.54 mmol/mol (−1.33%) in the icodec group and −12.57 mmol/ml (−1.15%) in the glargine U100 group, with a non-significant between-group difference in the change from baseline. Rates of level 1 hypoglycaemia were higher in the icodec group, with no significant differences in level 2 and severe hypoglycaemic episodes between the groups.

A subsequent open-label randomised clinical trial compared different icodec titration strategies with glargine U100 in 205 insulin-naive participants with type 2 diabetes over a 16 week period [24]. Initial doses were the same as in the previously described trial and insulin was titrated weekly in four different arms, including three icodec arms and one glargine U100 arm. Participants wore a continuous glucose monitoring (CGM) device (with blinded data) and the primary outcome was percentage time in range (TIR; 3.9–10.0 mmol/l). All groups achieved a similar percentage TIR, but level 2 hypoglycaemic episodes occurred at higher rates in the icodec groups with more aggressive titration protocols (groups B and C). The authors concluded that icodec titration A (blood glucose target 4.4–7.2 mmol/l with adjustment of ±21 U/week) displayed the best balance between glycaemic control and risk of hypoglycaemia.

Finally, the effects of switching from different basal insulin analogues to either icodec or glargine U100 were investigated in an open-label RCT including 154 participants with type 2 diabetes and a baseline HbA_1c_ of 53–86 mmol/mol (7.0–10.0%) [25]. The authors investigated whether a loading dose (i.e. daily analogue dose at baseline multiplied by seven and doubled, as first week dose) of icodec would lead to faster and better glycaemic control than no loading dose or glargine U100. Titration (±28 U/week for icodec vs ±4 U/day for glargine) was performed weekly based on pre-breakfast self-monitored blood glucose levels, with a target of 4.4–7.2 mmol/l blood glucose in all groups. The primary outcome was CGM-derived TIR. Participants receiving icodec with a loading dose achieved a higher percentage TIR than those receiving icodec without a loading dose or those receiving glargine U100 (72.9% vs 66.0% and 65%, respectively), with no significant differences between the groups in all grades of hypoglycaemia. The authors concluded that a loading dose could provide a higher percentage TIR without increases in rates of hypoglycaemic events.

#### Insulin BIF

The first Phase II study reported was an open-label RCT comparing switching from basal insulins to either BIF or degludec in individuals with type 2 diabetes [26]. The study included 399 participants randomised to BIF with a fasting glucose target of ≤7.8 mmol/l (group 1), BIF with a target of ≤6.7 mmol/l (group 2) or degludec with a target of ≤5.6 mmol/l (group 3). Interstitial fasting glucose measurements obtained with a CGM system were used for insulin titration. Both the loading and the weekly doses of BIF (expressed in mg) were based on the participants’ basal insulin dose prior to randomisation and the participants’ baseline HbA_1c_. The one-time loading dose ranged from 1.5–3 times the participants’ calculated weekly dose. Titration occurred every 2 weeks in group 1, every 4 weeks in group 2 and weekly in group 3. Oral glucose-lowering medications were continued throughout the study. Mean HbA_1c_ change from baseline was –6.56 mmol/mol (–0.6%) for both group 1 and group 2, and −7.7 mmol/mol (–0.7%) for degludec; BIF therefore had a similar efficacy to degludec. As per therapeutic target, fasting serum glucose levels were significantly higher among those treated with BIF, leading to a lower rate of hypoglycaemic episodes (both total and nocturnal) than with degludec.

The second Phase II study compared BIF with degludec in 278 insulin-naive participants with type 2 diabetes being treated with metformin with or without a DPP4i and/or sodium–glucose cotransporter 2 inhibitor (SGLT2i), with a fasting glucose target of 4.4–5.6 mmol/l [27]. HbA_1c_ reduction was comparable between groups at week 26, as was TIR, measured by blinded flash glucose monitoring. No severe hypoglycaemic episodes were reported. Numerically higher (without statistical significance) rates of level 1 hypoglycaemia were found in BIF-treated participants, with no difference between the groups in rates of level 2 or nocturnal hypoglycaemic episodes or in time below range.

The third Phase II study compared BIF with degludec in 265 participants with type 1 diabetes on multiple daily injections and receiving degludec, detemir or glargine before enrolment [28]. Both groups were titrated to a fasting blood glucose target of 4.4–5.6 mmol/l and all participants wore an unblinded CGM system. While HbA_1c_ levels were not statistically different, they were slightly higher in those treated with BIF [58 mmol/mol (7.50%)] than in those treated with degludec [57 mmol/mol (7.33%)] at week 26. This was accompanied by significantly higher fasting plasma glucose levels, particularly in the first weeks, in the BIF-treated group. This was probably related to the different titration target of 5.62–7.8 mmol/l for the first 2 weeks of treatment in the BIF group. No significant differences were found between the groups in rates of both level 1 and level 2 hypoglycaemic episodes.

**Fig. 2:**
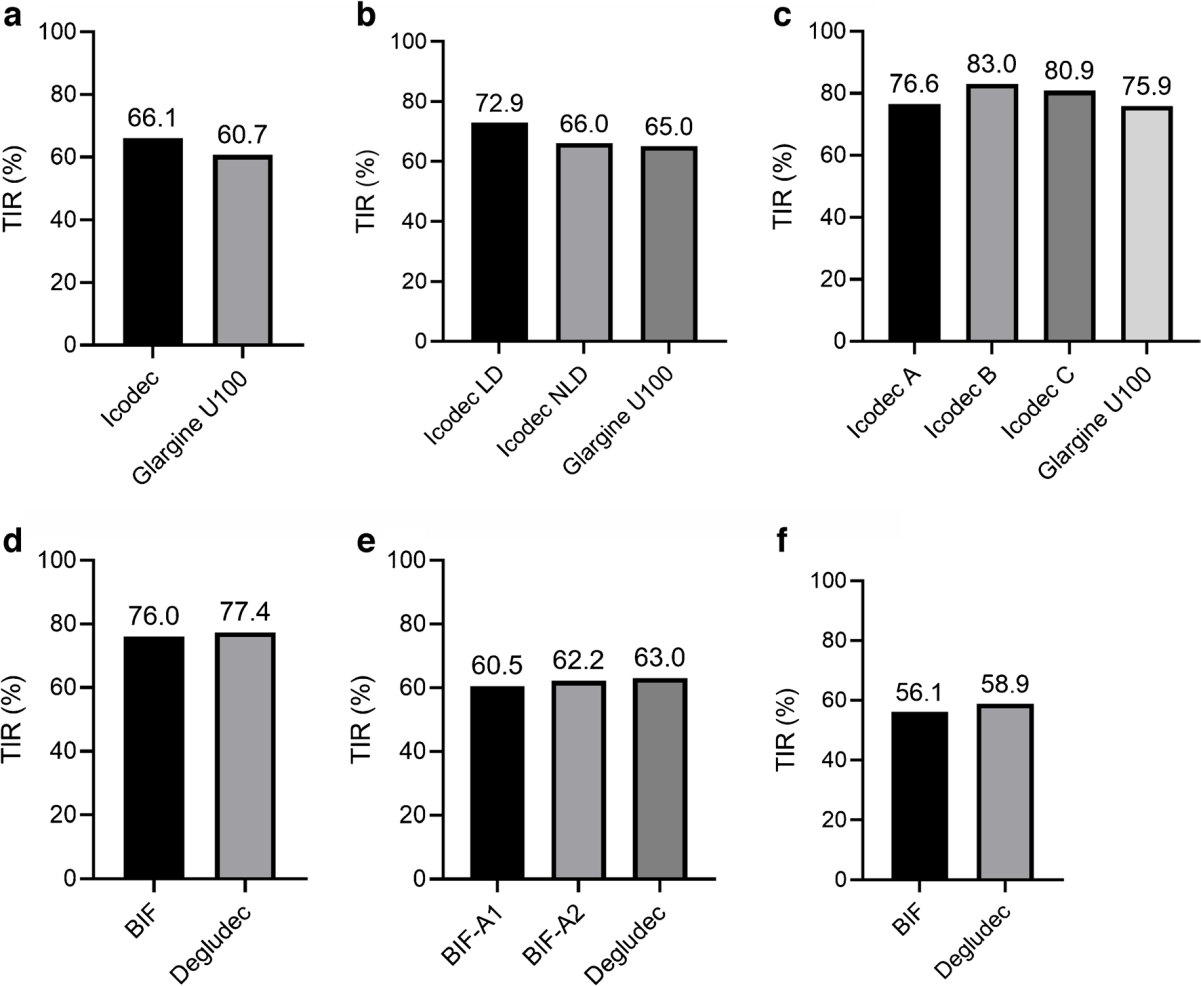
Proportion of TIR in Phase II clinical trials conducted with icodec (**a**–**c**) and BIF (**d**–**f**). (**a**) Insulin-naive participants with type 2 diabetes [23]; (**b**) insulin-treated participants with type 2 diabetes [25]; (**c**) insulin-naive participants with type 2 diabetes (titration study) [24]; (**d**) insulin-naive participants with type 2 diabetes [27]; (**e**) insulin-treated participants with type 2 diabetes [26]; (**f**) participants with type 1 diabetes [28]. TIR was defined as the proportion of time with glucose levels between 3.9 and 10 mmol/mol, except in the study by Rosenstock et al [23], in which a tighter range was chosen (3.9–7.8 mmol/l). LD, loading dose; NLD, no loading dose. This figure is available as part of a downloadable slideset

The features and results of the Phase II randomised clinical trials of icodec and BIF are shown in Table 1; the TIR achieved in each of these studies is shown in Fig. 2.

**Table 1:** Features and results of Phase II randomised clinical trials of weekly insulin analogues

Characteristic	Icodec			BIF		
	Rosenstock 2020 [23]	Bajaj 2021 [25]	Lingvay 2021 [24]	Frias 2023 [26]	Bue-Valleskey 2023 [27]	Kazda 2023 [28]
Features						
Design	Double-blind	Open-label	Open-label	Open-label	Open-label	Open-label
Duration	26 weeks	16 weeks	16 weeks	32 weeks	26 weeks	26 weeks
Participant	Insulin-naive participants with T2D	Insulin-treated participants with T2D	Insulin-naive participants with T2D	Insulin-treated participants with T2D	Insulin-naive participants with T2D	T1D participants on multiple daily injections
Comparator	Glargine U100	Glargine U100	Glargine U100	Degludec	Degludec	Degludec
Glucose targets (mmol/mol)	3.9–6.0 (both arms)	4.44–7.22 (both arms)	4.44–7.22 (icodec A and B and glargine U100); 3.89–5.99 (icodec C)	≤7.8 (BIF-A1); ≤6.7 (BIF-A2); ≤5.6 (degludec)	4.4–5.6 (both groups)	4.4–5.6 (both groups); 5.6–7.8 for BIF in first 2 weeks
No. of participants	247 (125 icodec, 122 glargine U100)	154 (54 icodec LD, 50 icodec NLD, 50 glargine U100)	205 (icodec A 51, B 51, C 52, glargine U100 51)	399 (135 BIF-A1, 132 BIF-A2, 132 degludec)	278 (143 BIF, 135 degludec)	265 (139 BIF, 126 degludec)
Baseline HbA_1c_, mean±SD						
mmol/mol	64±7.7	63±7.7	65±7.7	65±9.8	64±8.7	58±9.8
%	8.0±0.7	7.9±0.7	8.1±0.7	8.1±0.9	8.1±0.8	8.1±0.9
Baseline treatment	Metformin ± DPP4i	Basal insulin + metformin ± DPP4i/SGLT2i	Metformin ± DPP4i/SGLT2i	Basal insulin ± up to three non-insulin drugs	Metformin ± DPP4i/SGLT2i	Multiple daily insulin injections
Glucose monitoring	Flash glucose monitoring (weeks 25−26)	Dexcom G6	Dexcom G6	Dexcom G6	Libre Pro	Dexcom G6
Results^a^						
HbA_1c_, achieved, mmol/mol (%)	50 (6.69) (icodec) 52 (6.87) (glargine U100)	54 (7.1) (icodec LD) 57 (7.4) (icodec NLD) 57 (7.4) (glargine U100)	54 (7.1) (icodec A) 52 (6.9) (icodec B) 50 (6.7) (icodec C) 54 (7.1) (glargine U100)	60 (7.6) (BIF-A1) 57 (7.4) (BIF-A2) 57 (7.4) (degludec)	51 (6.8) (BIF) 50 (6.7) (degludec)	58 (7.5) (BIF) 57 (7.3) (degludec)
TIR (3.9–10.0 mmol/l) (%)	NA	72.9 (icodec LD) 66.0 (icodec NLD) 65.0 (glargine U100)	76.6 (icodec A) 83.0 (icodec B) 80.9 (icodec C) 75.9 (glargine U100)	60.5 (BIF-A1) 62.2 (BIF-A2) 63 (degludec)	76.0 (BIF) 77.4 (degludec)	56.1 (BIF) 58.9 (degludec)
Time in tight range (3.9−7.8 mmol/l) (%)	66.11 (icodec) 60.71 (glargine U100)	NA	NA	NA	NA	NA
Time below range (<3.9 mmol/l) (%)	NA	1.6 (icodec LD) 0.6 (icodec NLD) 0.5 (glargine U100)	1.0 (icodec A) 1.4 (icodec B) 1.9 (icodec C) 0.7 (glargine U100)	0.5 (BIF-A1) 0.7 (BIF-A2) 0.8 (degludec)	0.80 (BIF) 1.55 (degludec)	2.49 (BIF) 2.77 (degludec)
Body weight change (kg)	1.49 (icodec) 1.56 (glargine U100)	0.6 (icodec LD) 1.3 (icodec NLD) 0.1 (glargine U100)	0.9 (icodec A) 1.1 (icodec B) 1.3 (icodec C) 0.6 (glargine U100)	1 (BIF-A1) 1 (BIF-A2) 2 (degludec)	2.9 (BIF) 2.5 (degludec)	0.1 (BIF) 0.6 (degludec)
Level 1 hypoglycaemia (events/patient-year)	5.09 (icodec) 2.11 (glargine U100)	3.81(icodec LD) 4.29 (icodec NLD) 3.77 (glargine U100)	0.73 (icodec A) 1.11 (icodec B) 5.38 (icodec C) 0.58 (glargine U100)	23.0 (BIF-A1) 22.5 (BIF-A2) 30.0 (degludec)	3.3 (BIF) 2.8 (degludec)	207.6 (BIF) 206.7 (degludec)
Level 2 hypoglycaemia (events/patient-year)	0.53 (icodec) 0.46 (glargine U100)	0.78 (icodec LD) 0.15 (icodec NLD) 0.79 (glargine U100)	0.05 (icodec A) 0.15 (icodec B) 0.38 (icodec C) 0 (glargine U100)	2.2 (BIF-A1) 2.4 (BIF-A2) 3.0 (degludec)	0.22 (BIF) 0.15 (degludec)	40.7 (BIF) 45.5 (degludec)
Level 3 (severe) hypoglycaemia (events/patient-year)	0.01 (icodec) 0.02 0 (glargine U100)	0 (icodec LD) 0 (icodec NLD) 0 (glargine U100)	0 (icodec A) 0 (icodec B) 0 (icodec C) 0 (glargine U100)	0 (BIF-A1) 2 episodes (BIF-A2) 0 (degludec)	0 (BIF) 0 (degludec)	1 episode (BIF) 2 episodes (degludec)

^a^Data are mean values

LD, loading dose; NA, not available; NLD, no loading dose; T1D, type 1 diabetes; T2D, type 2 diabetes

### Ongoing Phase III programmes

While BIF is still being studied in larger scale Phase III RCTs, the icodec Phase III clinical trial programme has recently been concluded. The Phase III clinical trial programmes for icodec and BIF are known by the acronyms ONWARDS (ONce Weekly Analogue exploRing DiabeteS) and QWINT (Once Weekly Insulin Therapy), respectively. Here, we provide a brief overview of the study programmes and available results.

#### ONWARDS

The ONWARDS programme consisted of six RCTs performed in both individuals with type 2 diabetes (ONWARDS 1–5) and those with type 1 diabetes (ONWARDS 6). They were all open-label studies apart from ONWARDS 3, which was a double-blind RCT [29]. ONWARDS 5 featured real-world elements, including weekly insulin titration through a dosing app made available to participants and clinicians, and fewer site visits per protocol [30]. The other studies set a fasting blood glucose target of 4.4–7.2 mmol/mol, to be achieved with a weekly titration algorithm of ±20 U based on pre-breakfast blood glucose levels on the previous 3 days, in accordance with Phase II results [29, 31–34]. To decrease the risk of hypoglycaemia, sulfonylureas and glinides were suspended or at least reduced by 50% at the investigators’ discretion during the studies on participants with type 2 diabetes. The primary endpoint of all trials was HbA_1c_ reduction, while the main secondary outcomes included the percentage TIR, percentage of time spent with blood glucose levels <3.0 mmol/l or >10.0 mmol/l, number of clinically significant (level 2; <3.0 mmol/l glucose) or severe (level 3) hypoglycaemic episodes, change in fasting plasma glucose (FPG) and change in body weight [16, 35, 36]. The main features of the ONWARDS trials are summarised in Table 2.

**Table 2:** Summary of Phase III clinical programmes icodec (ONWARDS) and BIF (QWINT)

Trial	Design	No. of participants	Population	Comparator	Baseline treatment	Duration (weeks)
Icodec						
ONWARDS 1 [31]	Open label	984	Insulin-naive participants with T2D	Glargine U100	Any non-insulin drugs	78
ONWARDS 2 [32]	Open label	526	Insulin-treated participants with T2D	Degludec	Basal insulins ± non-insulin glucose-lowering agents	26
ONWARDS 3 [29]	Double-blind	588	Insulin-naive participants with T2D	Degludec	Any non-insulin drugs	26
ONWARDS 4 [33]	Open label	582	Insulin-treated participants with T2D	Glargine U100	Multiple daily insulin injections ± non-insulin drugs	26
ONWARDS 5 [30]	Open label	1085	Insulin-naive participants with T2D	Glargine U100/300 and degludec	Any non-insulin drugs	52
ONWARDS 6 [34]	Open label	583	Participants with T1D	Degludec	Multiple daily insulin injections	52
BIF						
QWINT-1 [38]	Open label	670	Insulin-naive participants with T2D	Glargine U100	At least one glucose-lowering medication	52
QWINT-2 [41]	Open label	912	Insulin-naive participants with T2D	Degludec	At least one glucose-lowering medication	52
QWINT-3 [42]	Open label	986	Insulin-treated participants with T2D	Degludec	Basal insulins ± up to three non-insulin drugs (except SUs)	78
QWINT-4 [40]	Open label	670	Insulin-treated participants with T2D	Glargine U100	Multiple daily insulin injections	26
QWINT-5 [39]	Open label	670	Participants with T1D	Degludec	Multiple daily insulin injections	52

SU, sulfonylureas; T1D, type 1 diabetes; T2D, type 2 diabetes

With regard to type 2 diabetes, these studies investigated icodec in both insulin-naive and insulin-experienced individuals. Active comparators were represented by degludec, glargine U100 or glargine U300, alone or in combination with aspart. HbA_1c_ reduction with icodec in those with type 2 diabetes was similar to that achieved with once-daily basal insulin analogues, but with a small benefit when icodec was compared with daily analogues without aspart. Furthermore, the superiority of icodec in terms of percentage TIR was achieved in ONWARDS 1 (the longest trial available to date [31]) compared with glargine U100; no differences between groups were observed in the other studies.

Regarding hypoglycaemic events, in those with type 2 diabetes, only ONWARDS 3 showed a statistically significant higher rate of clinically significant or severe hypoglycaemic events with icodec, albeit the rate remained below one event per patient-year of exposure (PYE) in both arms (0.32 in the icodec group, 0.12 in the degludec group; *p*=0.01) [29]. A significantly higher risk of hypoglycaemic events with icodec compared with degludec was identified in ONWARDS 6 in individuals with type 1 diabetes (19.93 events per PYE in the icodec group, 10.37 in the degludec group; *p*<0.0001), together with a longer time spent with blood glucose levels <3 mmol/mol [34]. Data on level 1 hypoglycaemia are available in ONWARDS 2 and 4, with higher risks of level 1 events found with icodec than degludec and glargine U100 plus aspart, respectively [32, 33].

There were no statistically significant differences between groups in terms of FPG reduction except for participants with type 1 diabetes in ONWARDS 6 (−0.56 mmol/l in the icodec group, −1.9 mmol/l in the degludec group; *p*<0.0001) [34]. No safety concerns emerged for icodec in terms of weight gain, with a statistically significant slight increase in weight compared with degludec seen only in ONWARDS 2 (+1.40 kg vs −0.30 kg; *p*=0.0004) [32]. Treatment satisfaction was assessed through the Diabetes Treatment Satisfaction Questionnaire (DTSQ) in three studies [30, 32, 34]. In participants with type 2 diabetes, a greater improvement in treatment satisfaction was reported in the icodec arms, while in those with type 1 diabetes the improvement was statistically higher for degludec. Participant compliance, assessed in ONWARDS 5 using the Treatment Related Impact Measure for Diabetes (TRIM-D) compliance domain score, was higher in the icodec arm [30]. Of note, among additional assessments, a greater percentage of participants with type 2 diabetes treated with icodec than with all active comparators achieved the target of HbA_1c_ <53 mmol/mol (7%) without level 2 or 3 hypoglycaemic events. This percentage was greater among participants with type 1 diabetes treated with degludec than treated with icodec in ONWARDS 6 [34].

A recent meta-analysis including both Phase II and Phase III trials conducted in individuals with type 2 diabetes showed that HbA_1c_ reduction was greater with icodec than with glargine and similar between icodec and degludec [37]. No significant differences between icodec and either glargine or degludec in rate of level 2 hypoglycaemic episodes were found.

**Fig. 3:**
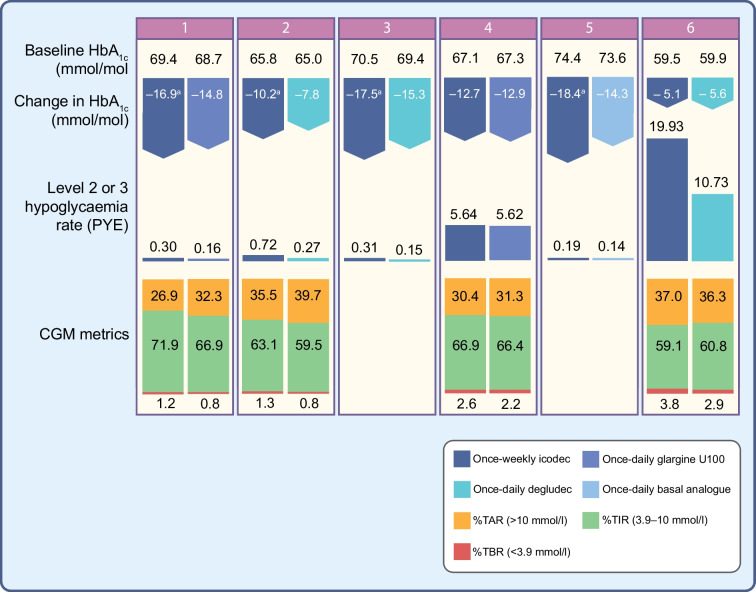
Main results of the Phase III ONWARDS programme evaluating the safety and efficacy of once-weekly icodec compared with once-daily glargine, degludec or a basal analogue in individuals with type 1 and type 2 diabetes. The numbers at the top of the figure correspond to the different ONWARDS trials (1–6 [29–34]). Features of the study populations included are shown in Table 2. ONWARDS 6 [34] included participants with type 1 diabetes. ^a^Statistically significant for superiority. TAR, time above range; TBR, time below range. This figure is available as part of a downloadable slideset

The main results of the ONWARDS programme RCTs are shown in Fig. 3.

#### QWINT

The QWINT programme consists of five open-label Phase III RCTs performed in both insulin-naive (QWINT-1 and -2) and insulin-experienced (QWINT-3 and -4) individuals with type 2 diabetes, and individuals with type 1 diabetes (QWINT-5) [38–42]. The primary endpoint for all trials is change in HbA_1c_ levels. The main features of these trials are summarised in Table 2. Active comparators consist of either degludec or glargine U100, alone or in a basal–bolus scheme. All of these RCTs are open label and, as of March 2024, no results from the programme are available.

### Making sense of the available studies

While several conclusions can be made from the available evidence, some pieces of the puzzle are still missing. Once-weekly insulins are effective. RCTs clearly show that they reduce HbA_1c_ to a similar, if not greater, extent than daily analogues. Similar results were found in flash glucose monitoring (FGM)/CGM studies, in which TIR was comparable between weekly and daily basal insulins, or even higher with weekly analogues. Once-weekly insulins also appear to be safe. There were no signs of specific reactions to the drugs in terms of injection site reactions, systemic reactions, tumourigenesis, excessive weight gain or production of antibodies against the molecules; moreover, severe hypoglycaemic episodes occurred at the same frequency as with once-daily basal analogues, at least in those with type 2 diabetes. This is promising, even though additional Phase III and IV studies with higher numbers of participants and longer follow-up times are needed to draw more definitive conclusions and exclude rare side effects. Safety and efficacy, together with the possibility of a higher degree of adherence in real-world settings (which was the main rationale supporting their development), make them a promising tool in the diabetologist’s armamentarium for diabetes treatment. This is even more the case if one considers the possibility of combining them with once-weekly glucagon-like peptide 1 receptor agonists (GLP1-RAs) to achieve target glycaemic control in both fasting and postprandial states with a single weekly injection. Indeed, RCTs comparing a weekly combination of icodec and semaglutide (IcoSema) with weekly and daily insulins are ongoing [43–45].

Higher rates of acceptance of and adherence to weekly analogues compared with daily comparators seem to be reinforced by DTSQ and TRIM-D results from icodec RCTs of individuals with type 2 diabetes [32]. Nonetheless, more evidence is needed to evaluate if and how the results from RCTs can be brought to real-life settings. Individuals enrolled in clinical trials are well-motivated and more likely to adhere to physicians’ prescriptions. This is also due, in part, to being able to attend frequent visits with expert physicians in tertiary care research centres. This aspect is particularly crucial given that available titration protocols, especially for BIF, are not straightforward. Efforts are needed to make titration as simple as possible both for physicians, who may initially feel disoriented by new numbers and algorithms, and for patients, who, in real life, will have to make adjustments on their own. Education of both patients and clinicians will make a difference in clinical practice. Concerning these issues, encouraging results have come from ONWARDS 5, in which fewer site visits with physicians were scheduled and titration was guided by a dosing app provided to participants, showing good efficacy, safety and adherence outcomes [30].

Another difference between participants enrolled in the RCTs described here and individuals in real life relates to the clinical features of the populations. Older individuals and those with a low eGFR and overt proteinuria were under-represented in the available studies. Older individuals and those with chronic kidney disease are at higher risk of hypoglycaemia and its sequelae [46, 47]. Moreover, overt proteinuria may influence insulin pharmacokinetics for icodec, as it may affect the circulating pool of the drug. Less stringent therapeutic targets might help to mitigate the hypoglycaemic risk in these individuals. Sub-analyses of Phase III RCTs involving thousands of participants will shed more light on these aspects.

At the present time, comparisons between the two once-weekly molecules, icodec and BIF, are problematic for several reasons. Studies differ in their design, active comparators and glycaemic targets to be achieved, both for the experimental arms and the comparator arms, and in their titration strategies (e.g. based on FPG or CGM data). Head-to-head RCTs, if performed, may provide more clear data on the relative safety and efficacy of the two molecules [48].

### Risk of hypoglycaemia

With regard to hypoglycaemia, the available evidence suggests a similar risk between once-weekly icodec or BIF and once-daily glargine or degludec in individuals with type 2 diabetes, while there are concerns of a higher risk of hypoglycaemia with icodec than degludec in those with type 1 diabetes [49]. It should be noted that comparisons between once-daily and once-weekly insulins are complicated by the use of different FPG targets between treatment arms (usually higher for weekly insulins) in most, although not all, available studies and by the different titration protocols followed. Given the longer half-lives of the once-weekly insulins, there were concerns over the possibility of long-lasting hypoglycaemic episodes with these molecules and whether these episodes would respond to usual oral carbohydrate therapy. Although the rates of level 2 and 3 hypoglycaemic events were not significantly higher than those experienced with once-daily insulins, at least for type 2 diabetes, it should be noted that, in all Phase III studies of type 2 diabetes, clinically relevant hypoglycaemic episodes (i.e. blood glucose <3 mmol/l) were frequently numerically higher in participants treated with icodec than in those treated with once-daily insulins [23, 26]. Moreover, in many instances, level 1 hypoglycaemic episodes occurred more frequently with once-weekly analogues. While these episodes may be less clinically relevant within an RCT, even grade 1 hypoglycaemia may alert individuals with diabetes and physicians in real-world settings.

Notably, hypoglycaemic episodes occurring in individuals enrolled in RCTs responded to usual oral carbohydrate corrective measures and the available evidence does not indicate that these hypoglycaemic episodes lasted longer than those reported with once-daily insulins [16]. A more detailed description of hypoglycaemic episodes derived from GCM also found similar lengths of episodes with once-weekly and once-daily insulins [50]. Even though the evidence from existing studies is promising, data from Phase III and real-world studies on the occurrence, duration and severity of hypoglycaemic episodes in different participant populations, as well as the effects of inadvertent or voluntary excessive insulin administration, are needed [51].

### Likely candidates for once-weekly insulins and potential uses

In the therapeutic algorithm for individuals with type 2 diabetes, once-weekly insulins are likely to be positioned as third-line drugs (after metformin and GLP1-RAs/SGLT2i) for those with uncontrolled HbA_1c_ levels, as once-daily basal insulins currently are [6]. Guidelines also suggest the use of insulin in cases of ongoing catabolism (weight loss), if symptoms of hyperglycaemia are present or when HbA_1c_ or blood glucose levels are very high [52]. While the use of once-weekly insulins in this specific setting has not been investigated, it is possible that a longer time period would be needed to achieve remission of symptoms, favouring once-daily insulins in this context (unless a loading dose is used to first achieve an adequate insulin concentration, as suggested for icodec). Use of daily analogues may also be considered for individuals admitted to hospital in a non-intensive care setting, where insulin demands may change rapidly based on the course of the underlying acute illness and the use of concomitant medications [52].

Possible candidates for once-weekly insulins are, in our opinion, individuals with type 2 diabetes followed at the outpatient clinic who do not achieve good glycaemic control with metformin, SGLT2i and/or GLP1-RAs or in whom there are contraindications or intolerance to these agents. In these cases, the need for fewer injections may also lead to higher rates of acceptance of and adherence to treatment. Similarly, those already taking once-daily insulins who experience difficulties with insulin injection and who worry about the complexity of their current regimen should be given the opportunity to switch to a once-weekly analogue. With regard to combining once-weekly insulins with other glucose-lowering drugs administered orally or by injection, generally the same rules used for once-daily insulins may be applied. Clinicians will have to learn to adjust the regimen of concurrent fast-acting insulin analogues in those on multiple daily injections and we strongly recommend against the combination of once-weekly insulins with glinides or sulfonylureas because of the added risk of severe hypoglycaemic events. Intriguingly, in ONWARDS 4, which focused on individuals with type 2 diabetes on a basal–bolus regimen, at the end of the study, while the total amount of insulin used was similar between the icodec and glargine U100 groups, participants in the icodec group received a significantly higher mean weekly basal insulin dose (305 vs 279 U) and a lower mean weekly bolus dose (197 vs 255 U) [33]. This might suggest better coverage of the whole day with the once-weekly analogue. We speculate that, in a real-world setting, where titration is less ambitious than in RCTs, aiming for a FPG level of around 7.2 mmol/l might lead to better overall glycaemic control if achieved with once-weekly analogues compared with once-daily formulations because coverage of the whole day is easily achieved, with a low absolute risk of severe hypoglycaemic events.

Finally, the use of once-weekly insulin may facilitate and simplify therapy in older individuals who depend on family members or other caregivers for insulin administration. In this case, the fasting blood glucose target must be increased and the titration algorithm must be less aggressive.

Data on type 1 diabetes are currently limited. Theoretically, a lower number of injections might prove useful in increasing adherence, especially in adolescence and young adulthood [53]. On the other hand, complete lack of feedback loops in insulin secretion leads to high glucose variability and rapidly changing insulin demands, which might not be met by a weekly insulin analogue. Moreover, the lower treatment satisfaction identified with insulin icodec in those with type 1 diabetes and the higher risk of hypoglycaemia, potentially resulting in safety issues, raise concerns and indicate the need for further investigation [34].

## Conclusions

In conclusion, the published and forthcoming data on once-weekly insulins are encouraging in terms of both efficacy for glycaemic control and risk of hypoglycaemia. More than 100 years after the introduction of insulin therapy, the ability to dramatically reduce the number of injections needed provides a great opportunity to simplify insulin therapy in many people with type 2 diabetes (and possibly also in those with type 1 diabetes who find it hard to accept new technologies and who have poor adherence to therapy). Appropriate education of physicians on the new weekly dosages and on adequate titration of the new molecules will be crucial. The simpler the instructions, the more likely it is that once-weekly insulins will be prescribed.

A more detailed analysis of the risk of hypoglycaemia with once-weekly insulins will also be essential for the safe use of these new molecules. In this regard, it should also be noted that in the real world the aggressive titration required by RCTs is not frequently applied, particularly in individuals with long-lasting diabetes and multiple comorbidities. A fasting glucose target above 7.2 mmol/l in these individuals should reduce the risk of hypoglycaemia. Finally, once-weekly insulins not only can reduce physicians’ therapeutic inertia, allowing a reduction in glucose load, but also may reduce the burden associated with diabetes and its complications.

### Supplementary Information

Below is the link to the electronic supplementary material.

Slideset of figures (PPTX 409 KB)

## Abbreviations

BIF: Basal insulin Fc
CGM: Continuous glucose monitoring
DPP4i: Dipeptidyl peptidase-4 inhibitor
DTSQ: Diabetes Treatment Satisfaction Questionnaire
FPG: Fasting plasma glucose
GLP1-RAs: Glucagon-like peptide 1 receptor agonist
ONWARDS: ONce Weekly Analogue exploRing DiabeteS
PYE: Patient-year of exposure
QWINT: Once Weekly Insulin Therapy
SGLT2i: Sodium–glucose cotransporter 2 inhibitor
TIR: Time in range
TRIM-D: Treatment Related Impact Measure for Diabetes
