# Supplementary material for: Once-weekly insulins: a promising approach to reduce the treatment burden in people with diabetes
Source: Diabetologia. 2024 Apr 29;67(8):1480–92. doi: 10.1007/s00125-024-06158-9 (PMC11343872; doi:10.1007/s00125-024-06158-9)
Supplement: Supplementary file 1 — Slideset of figures (PPTX 409 KB) [file 125_2024_6158_MOESM1_ESM.pptx]

## Slide 1
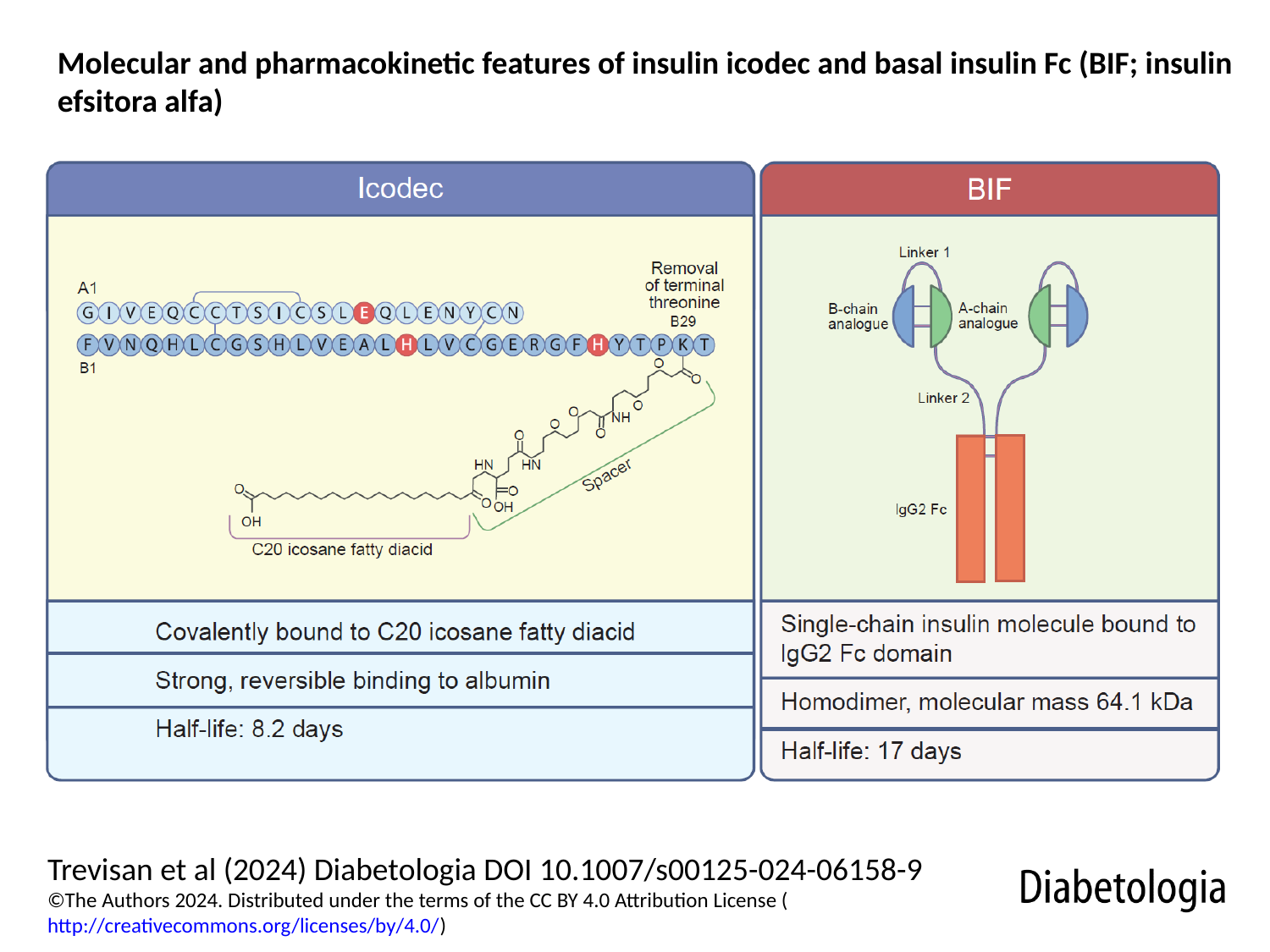

Molecular and pharmacokinetic features of insulin icodec and basal insulin Fc (BIF; insulin efsitora alfa)
Trevisan et al (2024) Diabetologia DOI 10.1007/s00125-024-06158-9
©The Authors 2024. Distributed under the terms of the CC BY 4.0 Attribution License (http://creativecommons.org/licenses/by/4.0/)

## Slide 2
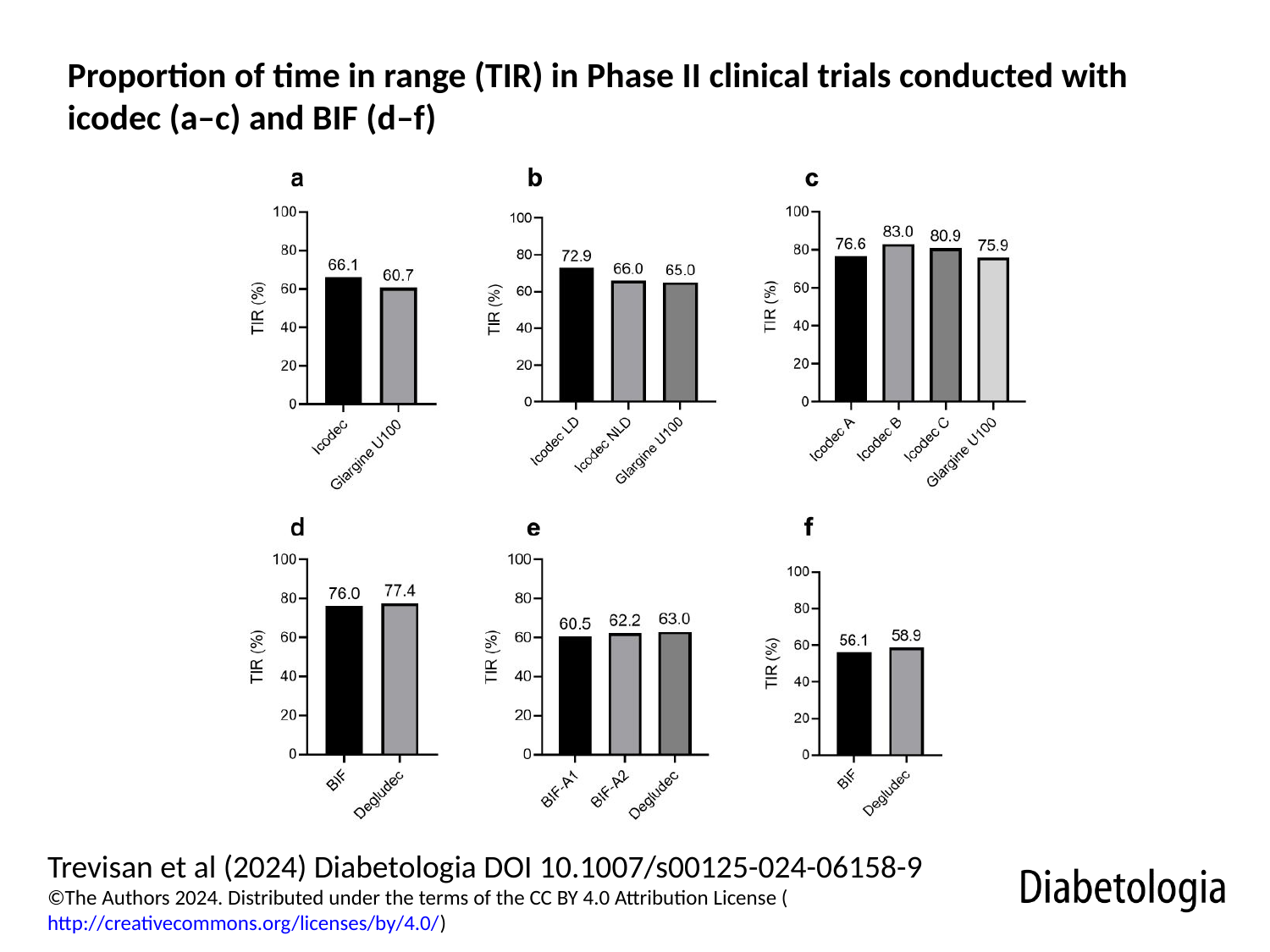

Proportion of time in range (TIR) in Phase II clinical trials conducted with icodec (a–c) and BIF (d–f)
Trevisan et al (2024) Diabetologia DOI 10.1007/s00125-024-06158-9
©The Authors 2024. Distributed under the terms of the CC BY 4.0 Attribution License (http://creativecommons.org/licenses/by/4.0/)

## Slide 3
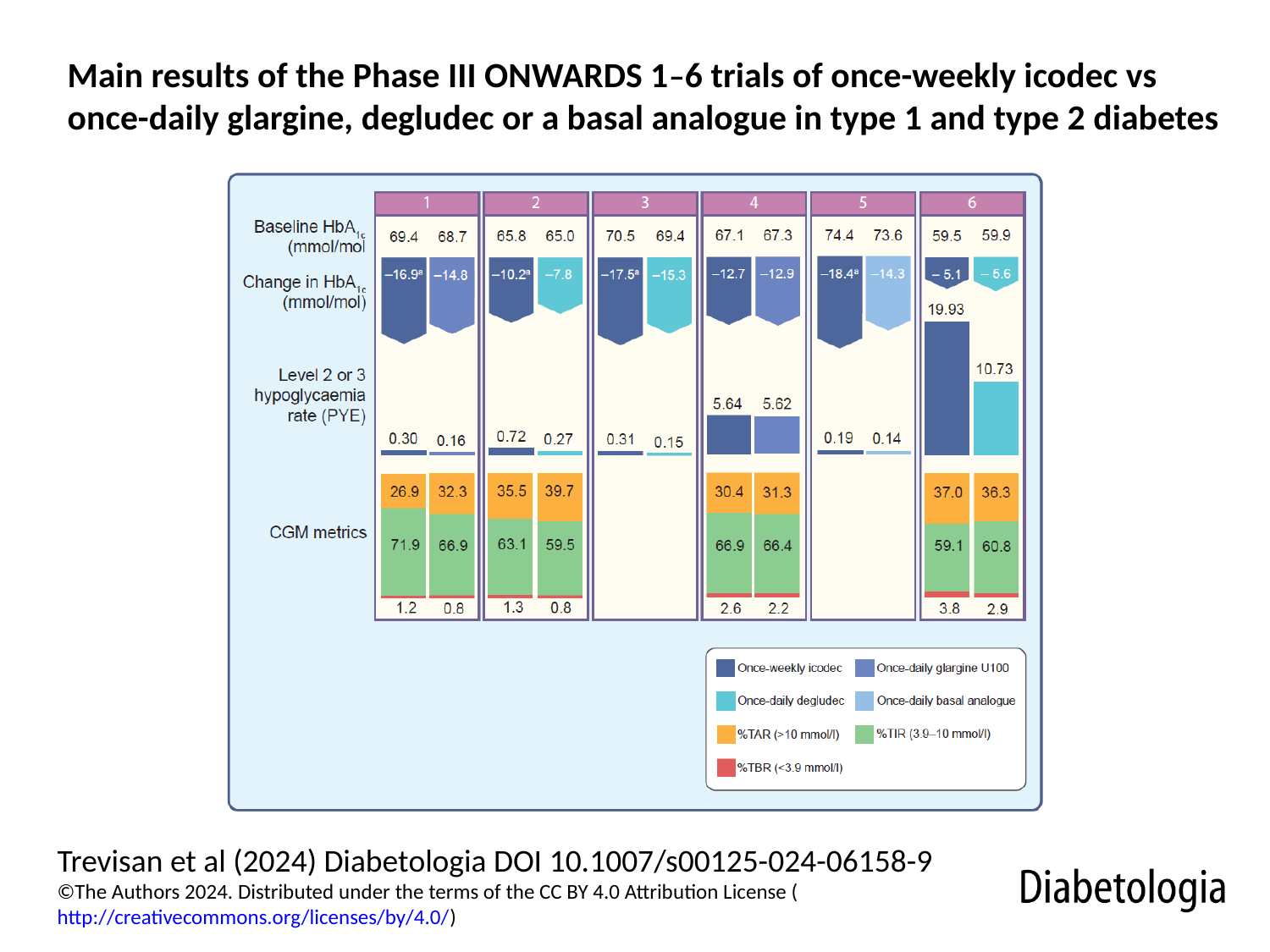

Main results of the Phase III ONWARDS 1–6 trials of once-weekly icodec vs once-daily glargine, degludec or a basal analogue in type 1 and type 2 diabetes
Trevisan et al (2024) Diabetologia DOI 10.1007/s00125-024-06158-9
©The Authors 2024. Distributed under the terms of the CC BY 4.0 Attribution License (http://creativecommons.org/licenses/by/4.0/)
